# Microwave-Assisted Synthesis and Characterization of Poly(*L*-lysine)-Based Polymer/Carbon Quantum Dot Nanomaterials for Biomedical Purposes

**DOI:** 10.3390/ma12233825

**Published:** 2019-11-21

**Authors:** Łukasz Janus, Marek Piątkowski, Julia Radwan-Pragłowska

**Affiliations:** Faculty of Chemical Engineering and Technology, Cracow University of Technology, 31-155 Kraków, Poland; marek.piatkowski@pk.edu.pl (M.P.); jrpraglowska@chemia.pk.edu.pl (J.R.-P.)

**Keywords:** carbon quantum dots, biocompatible nanomaterials, optical probes

## Abstract

Carbon nanomaterials in the form of quantum dots have a high potential due to their luminescent properties and low cytotoxicity which allows their use in optical probes for use in bioimaging and biodetection. In this article, we present a novel type of nanomaterials and their obtainment method under microwave-assisted conditions using poly(*L*-lysine) as a raw material. The ready products were characterized over their chemical structure, pH-dependent fluorescence properties and cytotoxicity on human dermal fibroblasts. Moreover, their antioxidant activity as well as ability to biologically active molecules (vitamins) and heavy metal ions detection was evaluated. The results confirmed the obtainment of biocompatible nanomaterials with advanced properties and good water solubility according to sustained development principles.

## 1. Introduction

In recent years great progress in the field of medicine and pharmacy has been observed, especially in the case of controlled drug delivery and release systems as well as diagnostics. Rapid development of nanotechnology provides many valuable solutions for doctors and their patients suffering from various diseases such as cancer or diabetes due to the extraordinary features of materials in the nano scale [1,2,3,4]. One of the most emerging nanomaterials are carbon quantum dots (CQDs) which are defined as particles of the size below 10 nm with a carbon core [1,2,3,4]. Although pure carbon is poorly soluble in water and has weak fluorescence abilities their unique characteristic include interesting properties such as tunable luminescence, high stability in water, and resistance to photobleaching and photoblinking. Contrary to many organic dyes or semiconductor quantum dots (inorganic nanomaterials), they are also quite soluble in aquatic solutions and are susceptible to various modifications. At the same time, they maintain their inertness to chemicals. Finally, they are biocompatible [4]. Therefore, carbon quantum dots are prior to most currently used photoluminescent substances and materials [5]. CQDs due to their low cytotoxicity may be applied in biomolecules/drugs delivery, biosensors manufacturing, and bioimaging. This class of nanomaterials can be also used in catalysis, optoelectronics, or optronics [1,5,6,7,8,9]. 

Carbon quantum dots were discovered in 2004 [4]. Since then numerous preparation pathways have been developed. There are two main approaches to their obtainment which are called “top-down” and “bottom up” [1,2,3,4,10]. The main preparation strategies include chemical or laser ablation, electrochemical digestion, microwave-assisted conditions, and finally solvothermal or hydrothermal pathway. However, there are some major problems to be considered during discussed nanomaterials obtainment. Firstly, during carbonization carbonaceous aggregation may occur. This can be omitted by applying confined pyrolysis, solution chemistry or electrochemical synthesis methods. Secondly, obtained nanodots can be non-uniform and of diverse sizes which leads to problems with their characterization and homogenous properties. This can be solved by applying post-treatment gel electrophoresis, dialysis, or centrifugation. Finally, very often the ready product surface properties such as water solubility, or selectivity are insufficient for their future applications [10,11,12,13,14,15].

CQDs synthesis can come along with their surface modification or may undergo post-reaction functionalization which helps with the aforementioned issues. It was proven that appropriate reaction conditions affect CQDs properties such as size, luminescent characteristics, cytotoxicity, or aquatic solutions solubility [1,2,3,4,15,16,17]. 

Carbon quantum dots are inexpensive in production and may be prepared from various sources. Chemical ablation which requires harsh conditions, as well as drastic processes, enables CQDs preparation as a result of small organic compound oxidation by strong acids into carbon nanomaterials. Electrochemical method is used to obtain C-nanodots from bulk carbon materials (graphite electrodes). Laser ablation of a carbon resulting in CQDs formation requires high temperature and pressure. The special attention is focused on nanomaterials prepared from carbon atoms rich waste biomass. Therefore, microwave-assisted and solvothermal/hydrothermal CQDs preparation methods can be considered as the most ecofriendly. Solvothermal pathway enables carbon nanodots obtainment as a result of a feedstock heating in high boiling solvents. The post-reaction mixture is subjected to extraction and concentration. Hydrothermal carbonization is a non-toxic procedure in which the solution of an organic compounds is placed in the sealed vessel and placed inside pressure reactor under high temperature. As a raw material, cellulose, chitosan, citric acid, glucose, or proteins were used. The main drawback is low reaction yield and long reaction times, typically 12 h. A great alternative to aforementioned methods is a microwave irradiation approach. Organic material subjected to microwaves turns into carbon nanomaterials in a few minutes [1,2,3,4,10]. 

The use of waste biomass as carbon quantum dots precursor results in the preparation of non-toxic materials, however very often there are characterized with unsatisfactory quantum yield. In many cases, very good fluorescent properties are faultily assigned to carbon quantum dots luminescent properties since the post-reaction mixture does not undergo purification and low-molecular by products which may be responsible for an intense fluorescence are not separated from CQDs. Such a phenomenon leads to misleading conclusions such as apparent cytotoxicity of the prepared materials [4,18,19,20].

To enhance fluorescent properties of the carbon quantum dots may undergo surface modification by the creation of covalent bonds, coordination, π–π interactions, and finally the sol–gel approach. Another method of luminescent characteristic improvement is doping with elements such as nitrogen, sulfur, or phosphor [4,16]. 

Fluorescence quantum yield is a very important parameter that allows achieving greater sensitivity and accuracy of optical probes [5,6,7,8,9]. 

Carbon quantum dots are reported to penetrate cell membrane due to their nano size. They may be used for bioimaging. CQDs are a powerful tool to describe various cellular processes in real time where biocompability of the material is crucial to maintain cell viability. Carbon nanodots may be also used to determine various ions and biomolecules levels and investigate cells metabolism. Bioimaging with the use of fluorescent compounds or nanomaterials is carried out using the appropriate apparatus and environment (cell culture medium). The use of fluorescent microscope may lead to the formation of the reactive oxygen species (ROS) which leads to cells apoptosis. Therefore, the antioxidant activity of the fluorescent dye used is a desired feature [21,22,23,24,25,26,27,28]. 

In this article, we present a novel, ecofriendly approach to the fast and efficient preparation of biocompatible carbon quantum dots applying poly(lysine) as a precursor under microwave-assisted conditions with the simultaneous nanomaterial modification. The ready bionanomaterials were investigated over their luminescent properties such fluorescence quantum yield and pH-sensitivity. Their chemical structure was determined by infrared (ATR-FTIR) spectroscopy. The obtained CQDs were studied over their ability of free radical removal. Finally, their ability to detect various biomolecules as well as metal ions was verified. The biocompatibility of the carbon quantum dots was confirmed by the XTT (2,3-Bis-(2-methoxy-4-nitro5-sulfophenyl)-2H-tetrazolium-5-carboxanilide salt) assay on primary cells–human dermal fibroblasts (HDF).

## 2. Materials and Methods 

### 2.1. Materials

*L*-lysine, propylene carbonate, NaOH, chitosan, HCl, DPPH (2,2-diphenyl-1-picrylhydrazyl) were all purchased from Sigma-Aldrich, Poznań, Poland. XTT (2,3-Bis-(2-Methoxy-4-Nitro-5-Sulfophenyl)-2H-Tetrazolium-5-Carboxanilide) assay, human dermal fibroblasts (HDF), and fibroblast growth medium (FGM) were also bought from Sigma-Aldrich, Poland. Multi-hole plates (96 holes) were baught from Nest, GenoPlast, Rokocin, Poland. 

### 2.2. Methods

#### 2.2.1. Poly(*L*-lysine)-Based CQDs Preparation 

To prepare precursor for CQDs synthesis 5 g of *L*-lysine and 15 mL of propylene carbonate were added into the reaction vessel. The reactants were mixed and the reaction vessel was placed into a Prolabo Synthewave 402 microwave (MW) reactor (Prolabo, Wrocław, Poland). The power of microwave radiation was set to 20% and time of polymerization reaction was set to 120 min and temperature 240 °C. During the polycondensation process, lysine was successfully dissolved in propylene carbonate. The ready product was cooled down. 

CQDs were prepared according to data presented in Table 1. Prolabo Synthewave 402 microwave reactor was used for carbonization of reaction mixture. After carbonization process, the aquatic solution of the product was diluted using distilled water followed by placing in an ultrasonic bath for 10 min (set temperature −50 °C). In the next step, the solution was purified from macro particles using standard filter paper. Then the solution was treated with sodium hydroxide (2 M) to achieve pH = 7 and separated from microparticles by membrane filters with the pore diameter of 0.2 µm. Finally, pre-purified solutions were separated from micro and nano leftovers of the low and high molecular weight non-carbonized poly(lysine) fragments using dialysis tubes (MWCO 500–1000, Sigma-Aldrich). The purification was carried out for four days with daily solvent exchange. To monitor the purification process, each day the UV–vis analysis was performed for the permeates. Fully purified nanodots were used for further investigations. 

#### 2.2.2. Carbon Quantum Dot FT-IR Anlaysis

The FT-IR analyses were conducted by FT-IR Nexus 470 Thermo Nicolet spectrometer (Thermo Fisher Scientific, Waltham, MA, USA) equippped with diamond ATR. To perform the analysis, each sample was fully dried to the form of powder. The range of the analysis was from 600 to 4000 cm^−1^. The no. of scans was 32. The resolution was set to 8 cm^−1^.

#### 2.2.3. Spectroscopic Properties Study of the CQDs

UV–vis analyses were performed with Agilent 8453 diode array spectrophotometer. Fluorescence measurements of CQD solutions were performed using a Jasco FP-750 (Jasco, Tokyo, Japan) spectrofluorimeter. The analyses were carried out applying quartz cuvettes (optical length = 1 cm). Fluorescence excitation was achieved by using an UV-light-emmiting xenon lamp at different excitation wavelengths. pH dependent fluorescence intensity emission of CQDs were performed by using citrate, phosphate, and ammonium buffer solutions (100 mmol) set to pH values of 4, 5, 6, 7, 8, 9, and 10 by using Elmetron CX-551 pH-meter with combined glass pH electrode. 

#### 2.2.4. Poly(lysine)-Based CQD Fluorescence Quantum Yield

The CQDs fluorescence quantum yield (Q) was calculated using Equation (1),

(1) $$Q_{s}=Q_{r}\frac{A_{r}}{A_{s}}\times \frac{E_{s}}{E_{r}}\times (\frac{\eta_{s}}{\eta_{r}}{)}^{2}$$

*Q* = Fluorescence quantum yield; *η* = Refractive index of the solvent; *A* = Absorbance of the solution; *E* = Integrated fluorescence intensity of emitted light. Symbols “*r*” and “*s*” refer to the reference and investigated samples respectively.

The *Q* value was investigated by parallel to quinine sulphate standard solutions dissloved in 0.1 M sulphuric acid solution, with a QY = 0.54 at 365 nm. 

#### 2.2.5. Carbon Quantum Dot Morphology Study by Transmission Electron Microscopy (TEM) Method

The microphotographs were taken using JEOL Transmission Electron Microscope (Jeol USA, lnc., Peabody, MA, USA). To perform analysis, each sample was dried and dissolved in methanol. In the next step, one drop of the solution containing CQDs was placed on the copper mesh covered with formvar and left to evaporate. The samples were evaluated using HT = 80000 V with exposure time 800 ms. Electron dose was 2771.9 e/nm^2^.

#### 2.2.6. Detection of Metal Ions and Vitamins

Metal ion solutions were prepared using twice-distilled water and NaCl, KCl, CaCl_2_, ZnSO_4_, HgCl_2_, MnCl_2_·4H_2_O, K_2_Cr_2_O_7_, and CuSO_4_·7H_2_O served as standards. Solutions of ascorbic acid and vitamin B6 were also prepared by using double distilled water. To prepare tests, a solution of CQDs carbon nanomaterials were membrane filtrated and mixed with the approptate amount of analytes solutions to get the concentration of metal ions on the level 100 mg/l and vitamin solution in the range 1–10 mg/mL. 

#### 2.2.7. Antioxidant Property Study of CQDs

To perform antioxidative properties study of CQDs a standard procedure with DPPH reagent was perfomed. A 25 mg/L solution of DPPH in 95% ethanol was prepared and used for all tests. To achieve a reliable results the prepared solutions of CQDs were dried overnight and weight of CQDs in solutions was determined. Measure of 0.2 mg/mL of all CQDs were prepared in twice-distilled water. To measure antioxidative properties 2 mL of DPPH solution was mixed with 1 mL of each CQDs solution respectively. The mixtures were keep in closed vials in darkness and the absorbance of blank and test solution was measured at 517 nm by using Agilent diode-array spectrophotometer. The antioxidative properties were calculated by comparision of the absorbance of blank and tested solutuions.

#### 2.2.8. Cytotoxicity Study

For the CQDs cytotoxicity evaluation human dermal fibroblasts (HDF) were used. CQDs solutions were sterilized using sterile membrane filters. For the study the primary cells were cultured at the temperature of 37 °C, high humidity (95%) and carbon dioxide 5% concentration. As a medium complete fibroblast growth medium (FGM) was applied. The medium was changed every 48 h in the 96-well plates. The cell culture lasted 5 days while the cell culture of HDFs in the presence of carbon nanomaterials duration was 48 h. The HDF morphology was investigated under inverted microscope (100×). To determine cytotoxicity of the prepared CQDs XTT (sodium 2,3,-bis(2-methoxy-4-nitro-5-sulfophenyl)-5-[(phenylamino)-carbonyl]-2H-tetrazolium) inner salt) assay was performed according to producer’s protocol. This test enables detection of the cellular metabolic activities through the color change from slightly yellow to orange. The absorbance was measured using UV–vis spectrophotometer at 475 nm. The dependence of concentration of CQDs vs. viability was determined by adding to the test solutions of different HDF amounts of CQDs to enrich concentrations of CQDs in the range of 0.005–0.040 mg/mL. 

## 3. Results

### 3.1. Fourier-Transform Infrared Analysis of the Prepared Carbon Qunatum Dots

**Figure 1:**
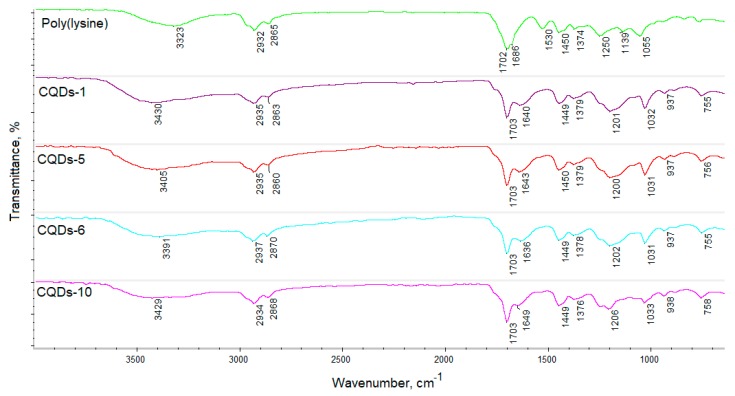
FT-IR spectra of the prepared CQDs.

Figure 1 shows infrared spectra of the poly(*L*-lysine) prepared by removing propylene carbonate from the reaction mixture under vaccum and obtained carbon quantum dots using aforementioned polymer as a precursor. It can be noticed that spectrum of the poly(aminoacid) shows broad band at 3323 cm^−1^ coming from hydroxylic groups of carboxyl croup and free amino groups present in the polymeric chain. The presence of bands typical for NH_2_ can be also noticed at 1530 cm^−1^. In the polymer structure, presence of C–H bonds confirms the presence of stretching vibrations present in the spectra at 2932 cm^−1^ and 2865 cm^−1^. Strong stretching vobration with a high absorbance present at 1702 cm^−1^ is present due to high concentration of –COOH functional groups in the sample. Moreover, band typical for amide bond can be observed at 1686 cm^−1^ what confirmes the amide bond formation in prepared polymer sample. The FT-IR spectra of the prepared CQDs are very similar and only small differences one may be observeed. Firstly, the intesity of bands coming from the functional groups (–COOH; –NH_2_) are much lower. It may be also noticed that there is a corellation between reaction time and chemical structure changes. It is known that irradiation time affects the size of the carbon core. As the duration of the process increases, the intensity of bands coming from carbonyl and amino groups decreases since polymeric chains undergo a carbonization process. It can be noticed, that the concentration of carboxylic and amine functional groups during chemical reaction decreases (in all series of samples). The strong band at 1703 cm^−1^ indicates that in CQDs high concentration of carboxylic groups is present. Bands present at 1031–1032 cm^−1^ indicates hydroxylic group presence in samples. Taking all into account, it can be concluded that the nanodots which arised from poly(*L*-lysine) are composed of the carbon core and polymeric chain residues. Thanks to the presence of hetermoatoms such as nitrogen, it may be assumed that the obtained nanomaterials will be characterized by very good luminescence properties [27]. The presence of hydrophilic functional groups, such as NH_2_ and COOH, is responsible for excellent water solubility comparing to traditional fluorescent organic dyes [4,21,22,23,24,25].

### 3.2. UV–Vis Analysis of the Prepared CQDs

**Figure 2:**
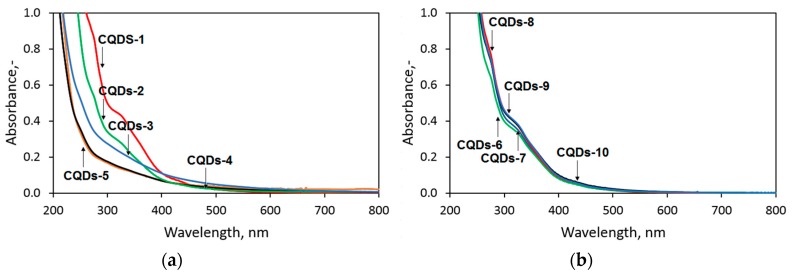
UV–vis characteristics of the prepared CQDs. (**a**) samples CQDs-1–CQDs-5; (**b**) samples CQDs-6–CQDs-10.

Generally, CQDs exhibit some characteristic absorption bands of different intensity. The first one is a π–π* electron transfer band appearing due to the presence of double C=C bonds in the carbon skeleton. The most important is a n–π* electron transfer which occurs if carbonyl or other groups are present in the sample [1,2,3,4]. The absorption of carbon nanodots is correlated with their luminescence properties. Fluorescence effects may depend on the internal and external surface of nanomaterials (different types of active fluorophores). The structure disorders appear due to the incorporation of heteroatoms such as nitrogen and sulphur. Therefore, carbon core functionalization or doping results in the enhancement of luminescence characteristics [27]. UV–vis spectra of the obtained nanomaterials are given in the Figure 2. It can be noticed that obtained spectra are characteristic for carbon quantum dots [4,12,21]. However, the absorption bands are different in the case of the samples with various irradiation duration and H_2_SO_4_ concentration used for the carbonization. It can be concluded that in the case of Samples 1–5 the crucial role in the carbonization process plays irradiation since a strong correlation between reaction time and absorption bands intensity is visible. Also, it may be noticed that samples CQDs-4 and CQDs-5 almost do not absorb strongly UV radiation in the range of 250–400 nm, which leads to the conclusion that nanodots consist only from carbon core whereas polymeric chain residues are poor in molecular fluorophores. On the other hand, Samples 6–10 exhibit almost identical absorbance which indicates that in the case of higher concentration of carbonizing agent (sulphuric acid), the mineral acid effect on carbonization becomes superior to microwave radiation. 

### 3.3. Fluorescence Characteristics of the Prepared CQDs

**Figure 3:**
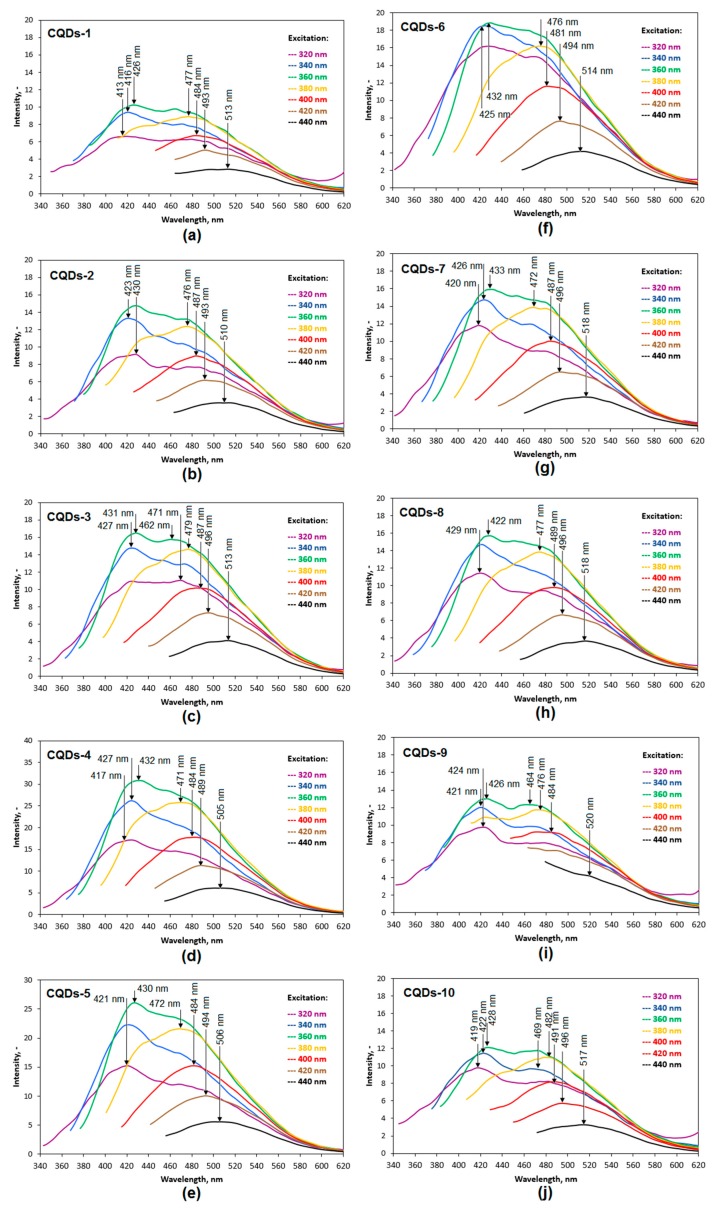
Fluorescence spectra of the prepared carbon quantum dots: (**a**) sample CQDs-1; (**b**) sample CQDs-2; (**c**) sample CQDs-3; (**d**) sample CQDs-4; (**e**) sample CQDs-5; (**f**) sample CQDs-6; (**g**) sample CQDs-7; (**h**) sample CQDs-8; (**i**) sample CQDs-9; (**j**) sample CQDs-10.

Carbon quantum dots have attracted a lot of attention thanks to their supreme luminescence properties, good cytocompability, and solubility in aquatic solutions which enables their application in medicine and pharmacy. Fluorescence of these materials can be explained by various factors. One of them is a disruption of graphite crystal structure, while the second one is presence of various chromophores on the surface of CQDs. Depending on the preparation method, CQD chemicals and physical structure can be modified to enhance desired properties. In this article, an attempt was made to obtain CQDs from poly(*L*-lysine) using various irradiation times as well as different mineral acid concentrations so to develop the most efficient obtainment strategy of fluorescent nanomaterials. The fluorescence spectra of the prepared CQDs are given in Figure 3. All of the samples can be considered as quantum dots since their spectra exhibit typical for CQDs shifts of the maximum peak to higher wavelengths depending on the excitation radiation energy [4,14,15,16,17,21]. It can be noticed that their fluorescence intensity is not uniform. The lowest fluorescence intensity is for CQDs-1 and CQDs-10, whereas the highest fluorescence intensity is observed for CQDs-4 and CQDs-5. Surprisingly, these results are in the opposite to UV–vis analysis results (Figure 2). Samples 2, 3, and 6–9 exhibit similar fluorescence properties. Taking into account synthesis parameters for each sample and obtained spectra it can be concluded, that in the case of the obtained samples a crucial role in the fluorescence effects plays both chromospheres and the core structure. Moreover, it is clearly visible that microwave irradiation time affect luminescence properties more than the carbonizing agent (sulphuric acid) which corresponds to the results presented in the Figure 3.

**Figure 4:**
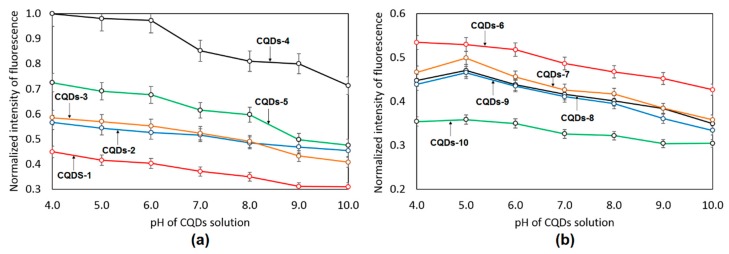
Fluorescence spectra of the prepared carbon quantum dots depending on pH value: (**a**) samples CQDs-1–CQDs-5; (**b**) samples CQDs-6–CQDs-10.

Nanomaterials for biomedical applications may be used in diagnostics, especially in the detection of various biomolecules and parameters monitoring cell cycles such as pH values [27]. Thus, pH-fluorescence sensitivity is a desired feature. The pH of most body fluids oscillates around 7. However, its decrease may suggest some molecular disfunctions or diseases. Figure 4 presents pH-sensitivity of the obtained CQDs. It may be observed that the nanomaterials exhibit fluorescence pH dependence. This property is especially noticeable in the case of samples CQDs-4 and CQDs-5. One may observe that nanodots emit the most intense fluorescence under pH below 6 and significant change of this parameter is observed when comparing to neutral pH. Such phenomenon can be explained by the presence of various functional groups on the dot surfaces. According to FTIR analysis and UV–vis analysis, it can be mainly caused by the protonation and deprotonation of amine and carboxylic acid functional groups [27]. 

#### Fluoresce Quantum Yield of the Prepared CQDs

**Figure 5:**
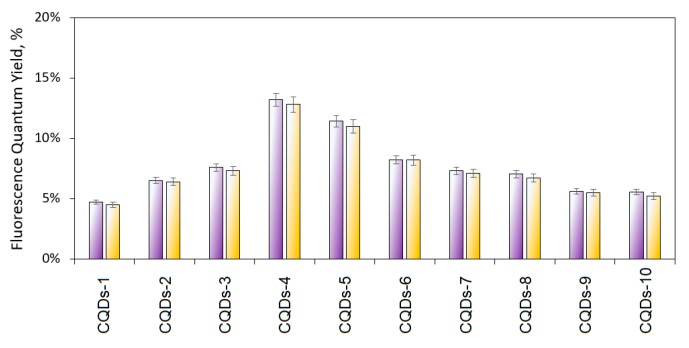
Fluorescence quantum yield of prepared CQDs. Violet bars—freshly after synthesis and purification. Yellow bars—fluorescence quantum yield after 2 months (photostability).

Fluorescence quantum yield is an important parameter for all substances and materials with potential luminescence properties and determines their applicability in various fields such as biosensors, optical probes, and bioimaging. There are multiple evidences that carbon quantum dots are characterized by high FQY. However, such results are often obtained due to the insufficient purification of the post-reaction mixture. It has been proven, that carbon dots based on lysine may reach quantum yield up to 23.3% [21]. Nevertheless, the authors performed dialysis of the raw product using 3500 Da MWCO and did not mention the time of the interferants removal. Thus, it is not known whether the high quantum yield comes from the carbon nanodots or low molecular weight polymeric oligomers. Fluorescence QY very often is determined with no purification at all thus the authors obtain misleading results and conclusions [23,24,25,26,27,28]. Figure 5 presents the results of fluorescence quantum yield determination and photostability. It may be observed that two samples (CQDs-4 and CQDs-5) are characterized by very good quantum yield, which can be assigned to the presence of the luminophores as well as the disturbance of the crystalline structure [2,4,11,12,13]. It can be observed that the results correspond to the data presented in Figure 3 and Figure 4. It is worth noting that the QY barely decreased over a two-month period. Thus, the obtained nanomaterials may be applied commercially due to their satisfactory shelf-live and very good photostability.

### 3.4. TEM Analysis of the Prepared CQDs

**Figure 6:**
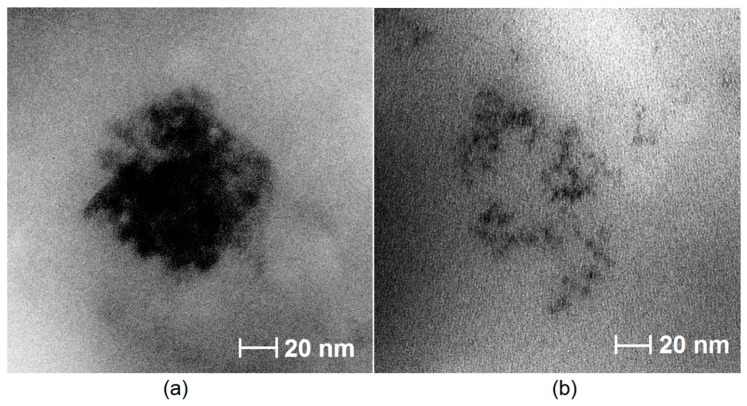
TEM images of the prepared CQDs: (**a**) CQDs-4; (**b**) CQDs-6.

Figure 6 presents TEM images of the samples which exhibited the highest quantum fluorescence yield (CQDs-4 and CQDs-6). It can be noticed that both types of carbon quantum dots are of the nano size below 10 nm. In the case of sample CQDs-4 (Figure 6a) the nanodots size is approximately 8–10 nm, while sample CQDs-6 (Figure 6b) contains nanodots of the size around 2–5 nm. The nanomaterials are of the spherical shape and their morphology are typical for carbon quantum dots [1,2,3,4]. It may be observed, that the carbon quantum dots size is strictly correlated with the synthesis parameters. It can be noticed that nanodots prepared using higher amount of chemical carbonizing agent (hydrochloric acid) are characterized by smaller size. What is interesting, in this case bigger nanodots have better fluorescence performance than quantum dots with lower diameters. It can be explained by the fact that using lower amount of carbonizing agent more functional groups (–COOH, NH_2_) are preserved.

**Figure 7:**
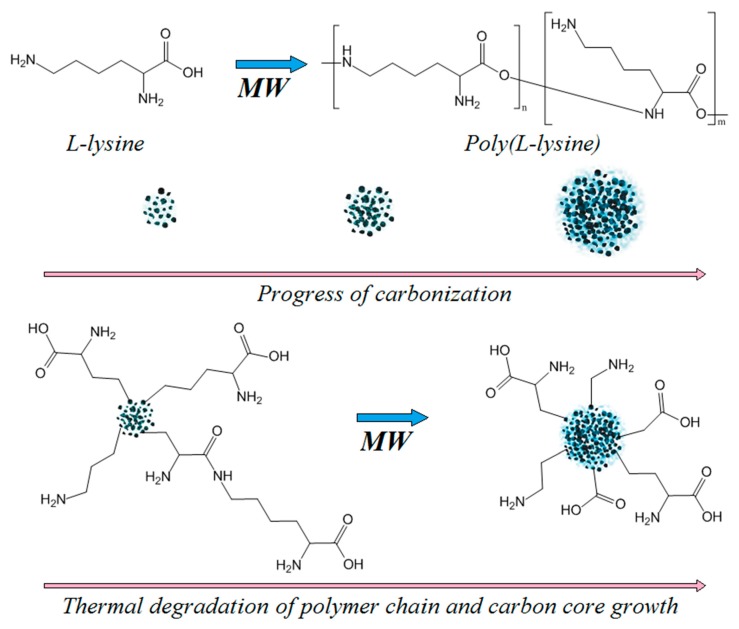
Schematic presentation of CQDs synthesis. Proposed chemical and morphological structure of the CQDs.

Figure 7 shows theoretical chemical and morphological structure of the nanodots which was proposed basing on the data collected from FT-IR analysis (Figure 1), TEM (Figure 6) images, and spectroscopic properties.

### 3.5. Detection Study of the Prepared CQDs

**Figure 8:**
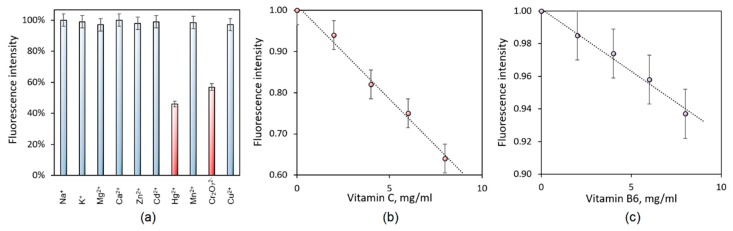
Detection of metal ions and vitamins by CQDs: (**a**) metal ions; (**b**) Vitamin C; (**c**) Vitamin B_6_.

Fluorescent dyes and materials are widely applied in various metal ions detection since they enable fast and efficient measurements. However, very often their effectiveness may be significantly decreased when detection is performed on the biological sample like blood or urine, since the presence of biological matrix components disturb the measurements. Figure 8 presents the results of metal ions detections. It can be noticed, that the CQDs-5 is able of selective detection of heavy metal ions (Hg^2+^, Cr^6+^). The results presented in Figure 8a show that nanomaterials change their fluorescence due to the interaction of the CQDs functional groups with different cations. The nanodots may coordinate certain ions. What is interesting, is that it may be observed that CQDs do not change their luminescence properties after contact with Na^+^, Mg^2+^, K^+^ ions which are a typical components of body fluids. Thus, it can be concluded that the nanomaterials can be applied for heavy ion detection in biological fluids without any time-consuming pre-treatment. Guo et al. reported preparation pathway of CQDs based on human hair applicable in metal ions detection [23]. He reports CQDs ability of mercury ions sensing which is possible due to the decrease in fluorescence intensity of 50%. However, our results show the decrease of this parameter of around 60% which suggests that proposed type of CQDs have better detection ability. What is more, Guo did not investigate the effect of biological matrix components (Na^+^, K^+^, Mg^2+^, etc.) so the real applicability of hair-based nanodots in biomedical applications has not been proven. Moreover, nanodots presented in this paper can also successfully detect chromium ions. Nanomaterials may be used in the detection of more complex structures such as proteins, vitamins, and others. Figure 8b,c presents results of the CQDs ability of molecules sensing performed using vitamins C and B_6_. The reaction of nanomaterials on vitamin presence may be a consequence in the fluorescence intensity decrease due to the electrostatic interactions between functional groups. Prepared carbon nanodots are rich in amino groups coming from poly(lysine). Figure 8b,c shows that CQDs can detect vitamin C, whereas the fluorescence quenching is less efficient in the presence of vitamin B6 which can be explained by the structures of both molecules. Vitamin C contains functional groups with negative charge which may interact electrostatically with the CQDs structure thus blocking chromospheres and disturbing luminescence emission. Thus, it may be concluded that the nanomaterials may be used for rapid detection of certain substances [18,28].

### 3.6. Antioxidant Properties Study

**Figure 9:**
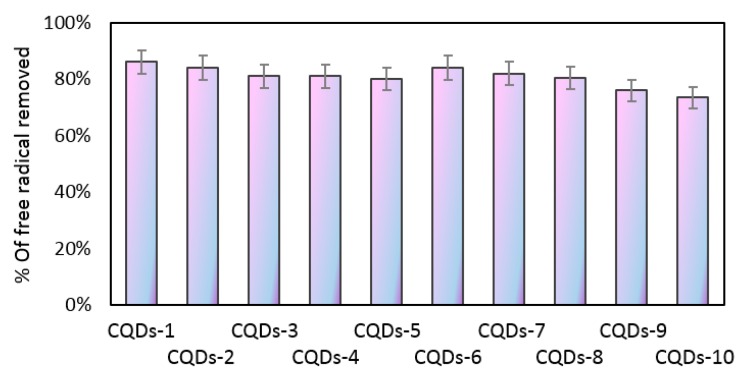
Antioxidant properties of the prepared CQDs against DPPH radicals.

Carbon quantum dots have a great potential in diagnostics and bioimaging due to their size below 10 nm, lack of cytotoxicity, and tunable luminescence properties. They can be used to detect various ions, biomolecules or to analyze cell metabolism and other molecular processes. However, to obtain reliable results the cell viability should be maintained. During imaging free radicals may be generated, especially reactive oxygen species leading to undesired cell behavior thus disrupting the analysis. Therefore, anti-oxidant activity of the nanomaterial used for the study is a highly desired feature. Figure 9 presents the results of antioxidant properties study by DPPH method. It can be observed that all samples have the ability of free radical scavenging. However, this feature depends strongly on the sample. It can be noticed that antioxidant properties are correlated with the synthesis parameters. The shorter the reaction time, the higher the amount of free radicals removed. It can be explained by the fact that during carbon dots formation, as the time of the reaction increases, the polymeric chain is transformed into the carbon core. As shown in Figure 1 the intensity of bands corresponding to the functional groups decreases with the reaction time. Therefore, it can be assumed that the shorter irradiation time results in the maintenance of functional groups such as carbonyl and amino which are responsible for free radicals removal. Taken all together, it can be assumed that prepared nanomaterials are able to protect viable cells from oxidative stress consequences, thus increasing the analysis efficiency and accuracy [23].

### 3.7. Cytotoxicity Study

**Figure 10:**
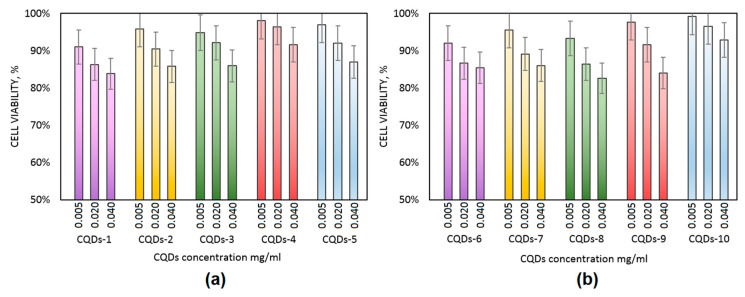
Results of the XTT assay on the prepared CQDs. (**a**) samples CQDs-1–CQDs-5; (**b**) samples CQDs-6–CQDs-10.

**Figure 11:**
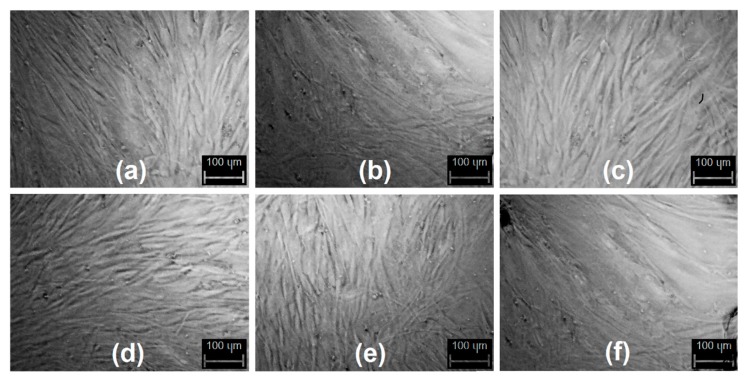
Images of the primary dermis cells (HDF) after 72h cell culture in the medium containing the CQDs. (**a**) sample CQDs-1; (**b**) sample CQDs-4; (**c**) sample CQDs-5; (**d**) sample CQDs-6; (**e**) sample CQDs-7; (**f**) sample CQDs-10.

Diagnostics development is crucial for early detection and successful diagnostics of cancer and other civil diseases [7,11,12]. Nanotechnology is a powerful tool which may be used in medicine and pharmacy since nano-scale particles are capable of cell membrane penetration. Carbon quantum dots which were obtained were evaluated for their biocompability with human dermal fibroblasts to verify their safety in biomedical applications as well as potential in diagnostics and bioimaging. Figure 10 presents the XTT test results. It can be noticed that all of the prepared samples are biocompatible according to ISO 10993-5:1999 standard since the number of viable cells comparing to the control is not lower than 17%. It can be observed that the toxic effect on primary cells is correlated with the CQDs concentration. No significant difference between different samples can be observed which can be assigned to the similar chemical composition of the CQDs solutions as shown at FT-IR spectra (Figure 1). Figure 11 presents microphotographs of the fibroblasts after 48 h of cell culture in the medium containing the CQDs solutions. One may observe that there are no abnormalities in cells morphology which are of spindle like shape. All cells are flattened. The HDF nuclei are oval and there are no grains in the cytoplasm. Such results confirm that CQDs were successfully purified and there are no toxic functional groups on their surface. Taking all of this into account, all of the data presented above indicates that the level of cytotoxicity of the prepared nanomaterials according to ISO 10993-5:1999 standard is 1 which confirms the biosafety of the CQDs.

## 4. Conclusions

In this article, we presented a novel method for advanced nanomaterials obtainment. The goal of the research was to prepare efficient polymeric-carbon quantum dots using poly(lysine) as a precursor according to green chemistry principles. The influence of microwave-irradiation time and carbonizing agent on CQD physicochemical and biological properties was evaluated. The nanodots’ chemical structures were characterized via infrared spectroscopy which showed that chemical structure of prepared CQDs depends from the carbonization conditions. The UV–vis and fluorescence spectra confirmed the obtainment of carbon quantum dots which have a very good pH-dependent fluorescence emission and stability. High fluorescence quantum yield enables the use of CQDs for optical probes for detection of mercury, dichromate, and vitamin C. Moreover, it was proved that the nanomaterials have antioxidant properties and are capable of molecules sensing due to the presence of certain functional groups on their surface such as amino and carboxyl. Finally, it was demonstrated that all of the prepared nanodots are not toxic to human primary cells according to ISO 10993-5:1999 standard. Therefore, it may be concluded that the prepared bionanomaterials have a great potential in medicine and pharmacy and may be applied successfully in optoelectronics.

## Figures and Tables

**Table 1:** Synthesis parameters of poly(lysine)/carbon quantum dot obtainment under MW radiation.

Entry	Poly(*L*-Lysine) in Propylene Carbonate, g	Amount of 35% H_2_SO_4_, mL	Reaction Time, min	MW Power, W
CQDs-1	0.40	0.20	1	300
CQDs-2	0.40	0.20	2	300
CQDs-3	0.40	0.20	3	300
CQDs-4	0.40	0.20	4	300
CQDs-5	0.40	0.20	5	300
CQDs-6	0.40	0.40	1	300
CQDs-7	0.40	0.40	2	300
CQDs-8	0.40	0.40	3	300
CQDs-9	0.40	0.40	4	300
CQDs-10	0.40	0.40	5	300

## References

[1] Wang Y., Hu A. (2014). Carbon quantum dots: Synthesis, properties and applications. J. Mater. Chem. C.

[2] Liang Z., Kang M., Payne G.F., Wang X., Sun R. (2016). Probing Energy and Electron Transfer Mechanisms in Fluorescence Quenching of Biomass Carbon Quantum Dots. ACS Appl. Mater. Interfaces.

[3] Yuan T., Meng T., He P., Shi Y.X., Li Y., Li X., Fan L., Yang S. (2019). Carbon quantum dots: An emerging material for optoelectronic applications. J. Mater. Chem. C.

[4] Janus Ł., Piątkowski M., Radwan-Pragłowska J., Bogdał D., Matysek D. (2019). Chitosan-Based Carbon Quantum Dots for Biomedical Applications: Synthesis and Characterization. Nanomaterials.

[5] Atabaev T. (2018). Doped carbon dots for sensing and bioimaging applications: A minireview. Nanomaterials.

[6] Kosowska M., Majchrowicz D., Ficek M., Wierzba P., Fleger Y., Fixler D., Szczerska M. (2020). Nanocrystalline diamond sheets as protective coatings for fiber-optic measurement head. Carbon.

[7] Hwang J.Y., Kim S.T., Han H.S., Kim K., Han J.S. (2016). Optical Aptamer Probes of Fluorescent Imaging to Rapid Monitoring of Circulating Tumor Cell. Sensors.

[8] Wojtkowski M., Szczerska M., Borycki D. (2010). Non invasive optical cellular imaging in humans. Photonics Lett..

[9] Marzejon M., Kosowska M., Majchrowicz D., Bułło-Piontecka B., Wąsowicz M., Jędrzejewska-Szczerska M. (2018). Label-free optical detection of cyclosporine in biological fluids. J. Biophotonics.

[10] Kandasamy G. (2019). Recent Advancements in Doped/Co-Doped Carbon Quantum Dots for Multi-Potential Applications. C—J. Carbon Res..

[11] Namdari P., Negahdari B., Eatemadi A. (2017). Synthesis, properties and biomedical applications of carbon-based quantum dots: An updated review. Biomed. Pharmacother..

[12] Molaei M.J. (2019). Carbon quantum dots and their biomedical and therapeutic applications: A review. RSC Adv..

[13] Zuo J., Jiang T., Zhao X., Xiong X., Xiao S., Zhu Z. (2015). Preparation and Application of Fluorescent Carbon Dots. J. Nanomater..

[14] Molaei M.J. (2019). A review on nanostructured carbon quantum dots and their applications in biotechnology, sensors, and chemiluminescence. Talanta.

[15] Abbas A., Mariana L.T., Phan A.N. (2018). Biomass-waste derived graphene quantum dots and their applications. Carbon.

[16] Liu X., Pang J., Xu F., Zhan X. (2016). Simple Approach to Synthesize Amino-Functionalized Carbon Dots by Carbonization of Chitosan. Sci. Rep..

[17] Chowdhury D., Gogoi N., Majumdar G. (2012). Fluorescent carbon dots obtained from chitosan gel. RSC Adv..

[18] Yoo D., Park Y., Cheon B., Park M.H. (2019). Carbon Dots as an Effective Fluorescent Sensing Platform for Metal Ion Detection. Nanoscale Res. Lett..

[19] Iannazzo D., Ziccarelli I., Pistone A. (2017). Graphene quantum dots: Multifunctional nanoplatforms for anticancer therapy. J. Mater. Chem. B.

[20] Das R.K., Mohapatra S. (2017). Highly luminescent, heteroatom-doped carbon quantum dots for ultrasensitive sensing of glucosamine and targeted imaging of liver cancer cells. J. Mater. Chem. B.

[21] Choi Y., Thongsai N., Chae S., Jo S., Kang E.B., Paoprasert P., Park S.Y., In I. (2016). Microwave-assisted synthesis of luminescent and biocompatible lysine-based carbon quantum dots. J. Ind. Eng. Chem..

[22] Pan M., Yin Z., Liu K., Du X., Liu H., Wang S. (2019). Carbon-Based Nanomaterials in Sensors for Food Safety. Nanomaterials.

[23] Guo Y., Zhang L., Cao F., Leng Y. (2016). Thermal treatment of hair for the synthesis of sustainable carbon quantum dots and the applications for sensing Hg^2+^. Sci. Rep..

[24] Rodríguez-Padrón D., Algarra M., Tarelho L.A.C., Frade J., Franco A., de Miguel G., Jiménez J., Rodríguez-Castellón E., Luque R. (2018). Catalyzed Microwave-Assisted Preparation of Carbon Quantum Dots from Lignocellulosic Residues. ACS Sustain. Chem. Eng..

[25] Chae A., Choi Y., Jo S., Paoprasert P., Park S.Y., In I. (2017). Microwave-assisted synthesis of fluorescent carbon quantum dots from an A2/B3 monomer set. RSC Adv..

[26] Huang C., Dong H., Su Y., Wu Y., Narron R., Yong Q. (2019). Synthesis of Carbon Quantum Dot Nanoparticles Derived from Byproducts in Bio-Refinery Process for Cell Imaging and In Vivo Bioimaging. Nanomaterials.

[27] Parvin S., Mandal T.K. (2016). Synthesis of Highly Fluorescence Nitrogen Doped Carbon Quantum Dots Bioimaging Probe, Their In vivo Clearance and Printing Applications. RSC Adv..

[28] Acha N.D., Elosúa C., Corres J.M., Arregui F.J. (2019). Fluorescent Sensors for the Detection of Heavy Metal Ions in Aqueous Media. Sensors.

